# Bogus Diplomas

**Published:** 1891-01

**Authors:** 


### BOGUS DIPLOMAS.

Through the courtesy of Mr. H. R. Slack, the efficient Secretary of the State Board of Pharmaceutical Examiners, the editor has had the opportunity of looking over two bogus medical diplomas, which have quite an interesting history. For several years previous to the late war there flourished in Alabama a pseudo-medical college chartered under the high-sounding appellation of the Graefenburg Medical Institute, whose president and entire faculty were embodied in the person of one Philip M. Shepperd. The building and accoutrements consisted of a log cabin in an old field, a skeleton and a few books and chemical reagents. Students who were not blessed with a superabundance of this world's goods paid their fees by working in the field. This institution graduated as many as fifteen or twenty full fledged M. D's. at one commencement, so we may safely presume that quite a number of its graduates have enjoyed all the legal rights and privileges vouchsafed to the graduates of the best medical colleges. Could there be a stronger argument for a revision of our existing laws, which allow all holders of diplomas to practice medicine? The clerks of the superior courts of the various counties are the sole judges of the reputability of the colleges from which the diplomas emanated. No stronger argument in favor of an efficient system of medical examiners could be offered.

